# *Panax ginseng* nanoemulsion for counteracting male infertility via modulating sex hormones and oxidative stress in a rat model

**DOI:** 10.1038/s41598-024-79388-x

**Published:** 2024-11-25

**Authors:** Basma I. El-Shimi, Rafat M. Mohareb, Hanaa H. Ahmed, Rehab S. Abohashem, Khaled F. Mahmoud, Demiana H. Hanna

**Affiliations:** 1 Chemistry Department, Faculty of Science, Cairo University, Giza, Egypt; 2 Hormones Department, Medical Research and Clinical Studies Institute, National Research Centre, Dokki, Giza, Egypt; 3 Stem Cell Lab, Centre of Excellence for Advanced Sciences, National Research Centre, Dokki, Giza, Egypt; 4 Food Technology Department, Food Industry and Nutrition Research Institute, National Research Centre, Dokki, Giza, Egypt

**Keywords:** Male infertility, Bisphenol A, Vitamin E, *Panax ginseng* dry extract nanoemulsion, Oxidative stress, Biochemistry, Developmental biology, Diseases, Nanoscience and technology

## Abstract

This study end to develop nanoemulsions of *Panax ginseng* dry extract and to evaluate the potential impact of these nanoemulsions *versus* free *Panax ginseng* dry extract and Vit.E in recovering male infertility induced in rats. Nanoemulsions of *Panax ginseng* dry extract were prepared by oil in water method. The designed samples were characterized by TEM, zeta sizer, FTIR, and TGA. The in vitro study included DPPH assay to estimate the free radical scavenging activity of the suggested treatments. The in vivo study included 100 adult male *Wistar* rats which were assigned into 10 equal groups; five groups of young rats weighting (150–200 g) and five groups of aged rats weighting (350–400 g). Group I, negative control. Group II, bisphenol-A (BPA). Group III, BPA+ *Panax ginseng* dry extract nanoemulsion. Group IV, BPA+ free *Panax ginseng* dry extract. Group V, BPA +Vit.E. After 40 days, serum total testosterone, free testosterone, MDA, 8-OHdG and AGEs were estimated. Besides, the histological investigation of testicular tissue sections was performed. TEM imaging of *Panax ginseng* dry extract nanoemulsions indicated spherical shape with diameter range from 2 to 50 nm, and the size distribution was in the range from 62 to 123 d.nm. The zeta potential of the designed nanoemulsions was -32.8 to -38.9 mV. FTIR spectra revealed the common active groups in the prepared nanoemulsions. The thermal stability of the nanoemulsions was up to 207 ºC. The in vitro results of DPPH assay showed % inhibition of DPPH free radical for *Panax ginseng* nanoemulsions samples was 49.38% (for young-treated group Sample A) and 72.28% (for aged-treated group Sample B), while for free *Panax ginseng* dry extract samples was 30.27% (for young-treated group Sample C) and 56.76% (for aged-treated group Sample D), for Vit.E samples was 32.36% (for young-treated group Sample E) and 36.39% (for aged-treated group Sample F).Thus the nanoemulsions exhibit free radicals scavenging activity more than free *Panax ginseng* dry extract and Vit.E. The in vivo findings elucidated that *Panax ginseng* dry extract nanoemulsions and Vit.E successfully revers the progressive insult of BPA on male fertility by significantly enhance total testosterone (2.87±0.318) and free testosterone (1.63±0.033) serum levels, and significantly decrease MDA (2.77±0.018), 8-OHdG (6.76±0.174) and AGEs (92.60±1.701) serum levels. Interestingly, the most promising outcomes were recorded upon the treatment with *Panax ginseng* dry extract nanoemulsions. In conclusion the developed *Panax ginseng* dry extract nanoemulsion could be used as a promising strategy in improving potential male infertility defects by rescuing male sex hormones, neutralizing oxidative stress and retrieving the structural organization of the testes.

## Introduction

Infertility is a medical condition affecting either male or female reproductive system, marked by the inability to achieve pregnancy following a year of consistent and unprotected sexual intercourse^1^. It represents a multifaceted crisis that commonly associated with psychological and emotional stress for both partners, as well as the significant economic burdens^2^. Infertility impacts approximately 15–20% of couples globally, with a male factor contributing to around half of these instances^3^. According to available data, certain regions like North Africa and the Middle East experience elevated rates of primary infertility prevalence, primary infertility refers to couples who have not had a live birth after at least 1 year having sex without using birth control methods^4^. In Egypt, a study conducted by the Egyptian Fertility Care Society and supported by the World Health Organization, revealed that infertility impacts 12% of Egyptian couples^5^.

Male infertility is known as the male inability to successfully impregnate a fertile female, following at least 12 months of unprotected sexual intercourse^6^. In the last three decades, an expanding body of research has elucidated the adverse effects of environmental, genetic, physiological, and lifestyle factors on fertility of male. Specifically, factors such as smoking, nutritional status, diabetes, psychological stress, overuse of alcohol/drugs, and endocrine disruptors have been highlighted. Research has demonstrated that these factors negatively impact sperm characteristics, DNA integrity and capacitation as well as the production of ATP by mitochondria^7,9^.

Bisphenol A (BPA) is a recognized endocrine disruptor renowned for its estrogenic properties^10^. It is crystalline chemical compound which extensively employed as a monomer in industrial processes for the manufacture of plastic materials, polyacrylates and polyesters^11^. Among its myriad applications, this compound is found in numerous everyday items, including linings for food and beverage containers, plastic tableware, kitchen tools, electronic devices (as cell phones and computers), dental sealants and thermal paper^12^.

Exposure to BPA elicits adverse effects on the male reproductive system, leading to disruptions in spermatogenesis and the quality of sperm^13^. Exposure to BPA results in a decrease in inhibin B levels, which correlates with a decline in the number of Sertoli cells, thus it directly impacts spermatogenesis^10^. Additionally, BPA causes a decrease in concentration of sperms, production of ATP and integrity of DNA^14^. BPA significantly diminishes both the motility and viability of epididymal sperm through the induction of oxidative stress^15^. In the study conducted by Eshak and Osman^16^, it was found that the reduced testosterone levels were linked to the elevated malondialdehyde (MDA) levels in rats treated with BPA. Even at the minimal concentrations, the estrogenic effects of BPA disrupt the functioning of the hypothalamic-pituitary-gonadal (HPG) axis^17^. Furthermore, low concentration of BPA diminishes testosterone levels by directly affecting Leydig cells, hindering their proliferation, and disrupting typical steroidogenesis^10^.

Korean red ginseng (*Panax ginseng*) is considered as a perennial herb, which is highly regarded in alternative and complementary medicines worldwide due to its numerous health-enhancing properties^18^. In traditional practices, ginseng root is utilized to augment male libido and fertility, while also elevating male sex hormone levels in various experimental models. Additionally, it enhances spermatogenesis, sperm count/vitality and restores Leydig/Sertoli cell count^19,20^. Lin et al.^21^ demonstrated that *Panax ginseng* stimulates testosterone synthesis and supports the proper functioning of androgens when administered as a dietary supplement. For an extended period, it has been observed that *Panax ginseng* exhibits protective characteristics against damage caused by free radicals^22^. Moreover, *Panax ginseng* is recognized for its established antioxidant and anti-inflammatory properties within the biological systems^23^.

Ginseng contains various groups of chemical components, such as ginsenosides, polysaccharides, phenolic acids, alkaloids and glucosides ^24^. Various reviews have reported that ginsenosides, which are the primary bioactive compounds in ginseng, could influence estrogen and androgen function and exert aphrodisiac effects^25^. The erectile response of the penis relies on maintaining equilibrium between vasoconstrictor agents, which induce contraction of cavernosal smooth muscle, thereby restricting blood flow, and vasodilator agents, which induce relaxation of cavernosal smooth muscle, thus facilitating increased blood flow and erection^26^. There is compelling evidence suggesting that ginsenosides can promote penile erection by directly stimulating vasodilation and relaxation of the penile corpus cavernosum^27^. Although ginseng exhibits promising pharmacological characteristics, its clinical utility is impeded by its inadequate bioavailability and poor solubility^28^. Nanobiotechnology has received considerable attention for its utilization of nanotechnology to address important medical and biological challenges as well as enhance the effectiveness of related applications^29^. Different nanodelivery systems, such as nanosuspensions, nanoparticles and nanoemulsions have recently gained attention for their role in improving numerous biophysical functionalities. This includes enhancing poorly soluble compounds dispersibility as well as providing sustained-release capabilities ^30^. The recent integration of ginseng into nanobiotechnology has led to the investigation of various ginseng-derived nanomaterials and their pharmacological effects, including antioxidant and anti-inflammatory properties^31,32^. Nanoparticles have exhibited favorable effects on fertility, as their smaller size facilitates their penetration of the testis. Nano-antioxidants have been noted for their ability to decrease reactive oxygen species (ROS), thereby effectively mitigating oxidative damage. Additionally, the nanoformulation of the antioxidant agents has been demonstrated to augment sperm motility and viability, alongside preserving DNA integrity and regulating gene expression levels ^33,34^. Vitamin E (Vit.E) is the general name of tocopherols, which are a group of phenolic chemical compounds^35^. It serves as one of the natural antioxidants present in semen, counteracting the actions of free radicals, inhibiting the generation of lipid peroxides, and shielding sperm from damage caused by ROS^36^. Vit.E opposes the detrimental effects induced by BPA on sperm parameters, testicular tissue, MDA and testosterone levels by enhancing the antioxidant enzymes activity and decreasing lipid peroxidation (LPO) ^37^. Additionally, it inhibits the initiation of apoptosis in spermatogenic, Leydig, and Sertoli cells^38^. The focus of our interest was to formulate nanoemulsion of *Panax ginseng* dry extract standardized to ginsenosides using oil in water method and to appraise the potent role of such nanoformulation against BPA-induced male infertility *via* hindering the oxidative stress process.

## Materials and methods

### Materials

#### Chemicals and drugs

Bisphenol A; 2, 2, -bis (4-hydroxyphenyl) propane, 4,4’- isopropylidenediphenol with purity ≥ 99% was purchased from Sigma-Aldrich Co-Saint Louis, Mo,63,103 − 2530 USA. Tween 20 (T20) used in the preparation of nanoformulation (stable oil-in-water emulsion) as an emulsifying agent, was procured from Sigma-Aldrich Co-Saint Louis, Mo,63,103 − 2530, USA. The standardized *Panax ginseng* dry extract (from roots of *Panax ginseng* C.A Meyer DER3-7:1) was purchased from Mulini, 6934 Bioggio, Switzerland. The content of ginsenosides, the pharmacological active ingredients of *Panax ginseng* dry extract, was 10% wt/wt. Vitamin E (Vit.E) was supplied from Pharco pharmaceuticals Alexandria, Egypt. The other commercially available reagents and chemicals were of analytical grade.

#### Animals

Adult male albino rats of *Wistar* strain were provided from the Animal Care Unit of the National Research Centre (NRC), Egypt. Rats was housed in a light/dark cycles of 12/12 h, a relative humidity of 55% ±5%, and a constant temperature of 22 ± 2˚C. Animals were allowed to have standard chow diet and water *ad libitum* throughout the experiment. Ethical Committee for Medical Research of the NRC, Egypt has been granted the final approval for the study under registration number of 13104112022.

## Methods

### Preparation of oil in water (O/W) *Panax ginseng* dry extract standardized to ginsenosides nanoemulsions

Nanoemulsion of *Panax ginseng* dry extract standardized to ginsenosides was prepared using the oil-in-water system according to Min et al.^39^ with some modifications. In brief, 4 mg of ginsenosides (equivalent to 40 mg of *Panax ginseng* dry extract) was dissolved in 0.75 ml deionized water for preparation of the dose for young adult rats (Sample A), and 7 mg of ginsenosides (equivalent to 70 mg of *Panax ginseng* dry extract) was dissolved in 0.75 ml deionized water for preparation of the dose for aged adult rats (Sample B). Then Tween 20 was added to the mixture gradually, the mixture was homogenized to form nanoemulsions by a high speed homogenizer (Model: 400ELPC, PRO Scientific Inc., 01-02411ELPC homogenizer, USA) at 20,000 rpm for 8 min. in ice bath to reduce the temperature of the mixture. After that, the emulsions were sonicated by ultrasonicator (Ultrasonic Cleaner MTI Corporation, Model UD150SH3.8LQ, USA) at 750 W for 5 min. for dispersion of particles from each other. Finally, portion of both nanoemulsion samples were subjected to freeze drying using the freeze drier (LABCONCO, FreeZone 12 L freeze-drying system with stopper tray, catalog number: 7759030) to transform nanoemulsion into powder for measuring Fourier transform infrared (FTIR) spectroscopy and Thermogravimetric analysis (TGA).

### Characterization of nanoemulsions of *Panax ginseng* dry extract

All characterization tests were done at the Central Lab of the NRC, Egypt. *Panax ginseng* dry extract standardized to ginsenosides nanoemulsions were characterized using transmission electron microscope (TEM) Model (JEM-2100, JEOL, Japan) at an accelerating voltage of 200KV. The prepared samples were diluted with distilled water and sonicated for 3 min. in an ultrasonic bath (4HT10146 by CREST ULTRASONICS, Trenton, NJ, USA), then 5 µl from each sample were taken by micropipette and placed on a film-coated 200-mesh copper specimen grids for 10 min. and the excess fluid was eliminated using the filter paper. Finally, the grids were subjected to TEM. The polydispersity index (PDI), zeta sizer and zeta potential of the prepared samples was detected by using Malvern Zetasizer (Nano-ZS, Malvern Instrument, UK). Each sample was dispersed in distilled water under ultra-sonication for 10 min. The size distribution and zeta-potential for each sample were measured using the Malvern zeta sizer Nano-ZS, Malvern Instrument with He/Ne laser at wavelength λ = 633 nm and an angle of 173° using collecting backscatter optics^40^. Fourier transform infrared (FTIR) spectroscopy was used to determine the functional groups of the prepared samples. A small amount (3.5 mg) of each sample was placed directly on the lens of FTIR (Bruker VERTEX 80, Germany) combined with Platinum Diamond ATR to comprise a diamond disk as an internal reflector in the range 4000–400 cm^− 1^ with resolution of 4 cm^− 1^, and refractive index of 2.4 ^41^. The thermogravimetric analysis (TGA) was used to detect the thermal stability of the prepared samples. The samples were heated at 10 °C/min in the atmosphere of nitrogen (SETARAM Instrumentation, model Themys one plus, France) for 22.5 min.

### Determination of free radical scavenging capacity in vitro using DPPH assay

The 1,1 - diphenyl − 2 - picrylhydrazyl (DPPH) radical assay was carried out spectrophotometrically (Shimandzu, Tokyo, Japan) to estimate the free radical scavenging activity for samples A (*Panax ginseng* dry extract nanoemulsion young group), B (*Panax ginseng* dry extract nanoemulsion aged group), C (free *Panax ginseng* dry extract young group), D (free *Panax ginseng* dry extract aged group), E (Vit.E young group), and F (Vit.E aged group). A volume of 50 µl of each sample was combined with 5 ml of a 0.004% ethanol solution of DPPH. Following an incubation period of 30 min. at room temperature, the absorbance was measured against a control (contains all reagents except the tested sample and ascorbic acid) at 517 nm. Ascorbic acid (AA) served as a reference standard and was dissolved in methanol to prepare a stock solution with a concentration of 1 mg/ml.

$$\:\text{I}\:\left({\%}\right)=\frac{(Abs.\:of\:control-Abs.\:of\:sample\:or\:standard)}{Abs.\:of\:control}\times\:100$$

Where: Abs. of control is the absorbance of the control reaction (contains all reagents except the tested sample and ascorbic acid) and Abs. of sample/standard is the absorbance of the tested sample. I (%) is the inhibition percentage of DPPH radicle. This test was performed in triplicate for validation of the results.

### Animal experiment

This study was conducted on one hundred adult male albino rats of *Wistar* strain which were assigned as follows:

fifty young rats weighting 150–200 g (3 month-old) and fifty aged rat weighting 350–400 g (12 month-old), that were randomly classified as:

negative young control group (young control group) that was orally administered with corn oil (1 ml/rat), negative aged control group (aged control group) that was orally administered with corn oil (1 ml/rat), BPA young group (BPA young-challenged group) that was orally administered with 250 mg/kg/day of BPA dissolved in corn oil according to the study of Malmir et al.^42^, BPA aged group (BPA aged-challenged group) that was orally administered with 250 mg/kg/day of BPA dissolved in corn oil, BPA + *Panax ginseng* dry extract nanoemulsion young group (Nano young-treated group) that was orally administered with 250 mg/kg/day of BPA dissolved in corn oil simultaneously with intraperitoneal administration of *Panax ginseng* dry extract nanoemulsion (20 mg/kg/day Sample A), BPA + *Panax ginseng* dry extract nanoemulsion aged group (Nano aged-treated group) that was orally administered with 250 mg/kg/day of BPA dissolved in corn oil simultaneously with intraperitoneal administration of *Panax ginseng* dry extract nanoemulsion (20 mg/kg/day Sample B), BPA + free *Panax ginseng* dry extract young group (Free young-treated group) that was orally administered with 250 mg/kg/day of BPA dissolved in corn oil simultaneously with intraperitoneal administration of *Panax ginseng* dry extract dissolved in saline (20 mg/kg/day Sample C) according to method of Kim et al.^43^, BPA + free *Panax ginseng* dry extract aged group (Free aged-treated group) that was orally administered with 250 mg/kg/day of BPA dissolved in corn oil simultaneously with intraperitoneal administration of *Panax ginseng* dry extract dissolved in saline (20 mg/kg/day Sample D), BPA + Vit.E young group (Vit.E young-treated group) that was orally administered with 250 mg/kg/day of BPA dissolved in corn oil simultaneously with oral administration of Vit.E (150 mg/kg/day Sample E) dissolved in corn oil according to Malmir et al.^42^, BPA + Vit.E aged group (Vit.E aged-treated group) that was orally administered with 250 mg/kg/day of BPA dissolved in corn oil simultaneously with oral administration of Vit.E (150 mg/kg/day Sample F) dissolved in corn oil.

The administration of BPA as well as the different treatments were implemented at the same time and the experiment lasted for 40 days.

### Biochemical analyses

After finalizing the experiment (40 days), the rats in different studied groups were fasted overnight and anaesthetized with pentobarbital 45 mg/kg intraperitoneally^44^ to collect blood samples from the tail vein. Then, the animals were sacrificed and the testes were dissected and washed in saline solution, then fixed in formalin saline (10%) for histological investigation. The serum samples were separated by centrifugation of blood samples at 1800 x g (times gravity) for 10 min. at 4˚C. The serum samples were kept at -20˚C pending biochemical determinations which included total testosterone, free testosterone, 8-hydroxy-2′-deoxyguanosine (8-OHdG) and advanced glycation end products (AGEs) that were measured by enzyme linked immunosorbent (ELIZA) using ELIZA kits (Sunlong, China, Catalogue Number SL1061Ra, SL0293Ra, SL0019Ra, SL0036Ra respectively) according to the operating’s instructions. Serum MDA level was quantified by colorimetric method using a colorimetric kit (Biodiagnostic, Egypt, Catalogue Number MD 25 29) following the manufacturer’s manual^45^.

### Histological examination

After fixation of the testicular tissue in 10% formalin saline for 24 h, the tissue samples underwent washing with tap water before being prepared and stained for light microscopy (Leica, Wetzlar, Germany). The process involved dehydrating the fixed tissue through a series of ethyl alcohol dilutions (ranging from 70 to 100%) and subsequently clearing the specimens in xylene. The tissue was then embedded in paraffin wax using a hot air oven set at 56 °C for 6 h. Following this, paraffin wax tissue blocks were sectioned using a microtome (Leica RM2255, Wetzlar, Germany) at a thickness of 5–6 microns. The resulting sections were collected on glass slides, deparaffinized, and stained with hematoxylin and eosin for routine histological examination under a light microscope^46^.

#### Statistical analyses

In the present study, all results were expressed as Mean ± S.E (Standard Error) of the mean. The data were analyzed by one-way analysis of variance (ANOVA) using the Statistical Package for the Social Sciences (SPSS) program, version 20 followed by least significant difference (LSD) test ^47^. Difference was considered significant at *P* < 0.05. The percent of change between groups was calculated according to the equation:

$$\:{\%}\:of\:change=\left(\frac{mean\:of\:treated\:group-meanof\:control\:group}{mean\:of\:control\:group}\right)\times\:100$$

## Results

### Characterization of nanoemulsions of *Panax ginseng* dry extract (sample A and sample B)

#### Transmission electron microscope (TEM)

**Fig. 1:**
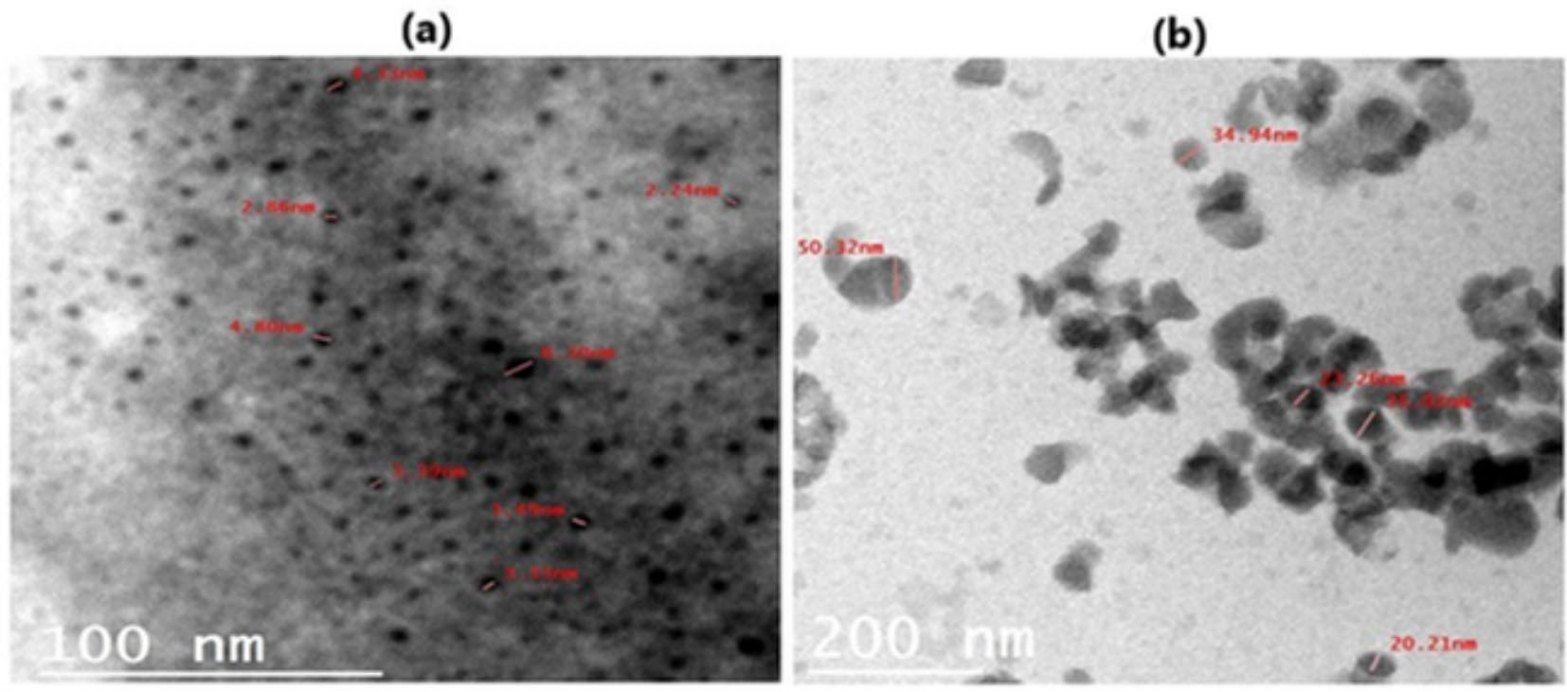
TEM micrograph of sample A (**a**) showing a completely spherical shape of particles and sample B (**b**) showing spherical shape inclined to hexagonal shape of the particles.

The obtained data from TEM for samples A and B showed that: sample A of a completely spherical shape of the particles with a diameter ranges from 2 to 8 nm and homogenous distribution (Fig. 1a). Whereas, sample B showed a spherical shape of the particles inclined to hexagonal shape with a diameter ranges from 20 to 50 nm, and heterogeneous distribution (Fig. 1b).

#### Polydispersity index (PDI), zeta sizer and zeta potential

**Fig. 2:**
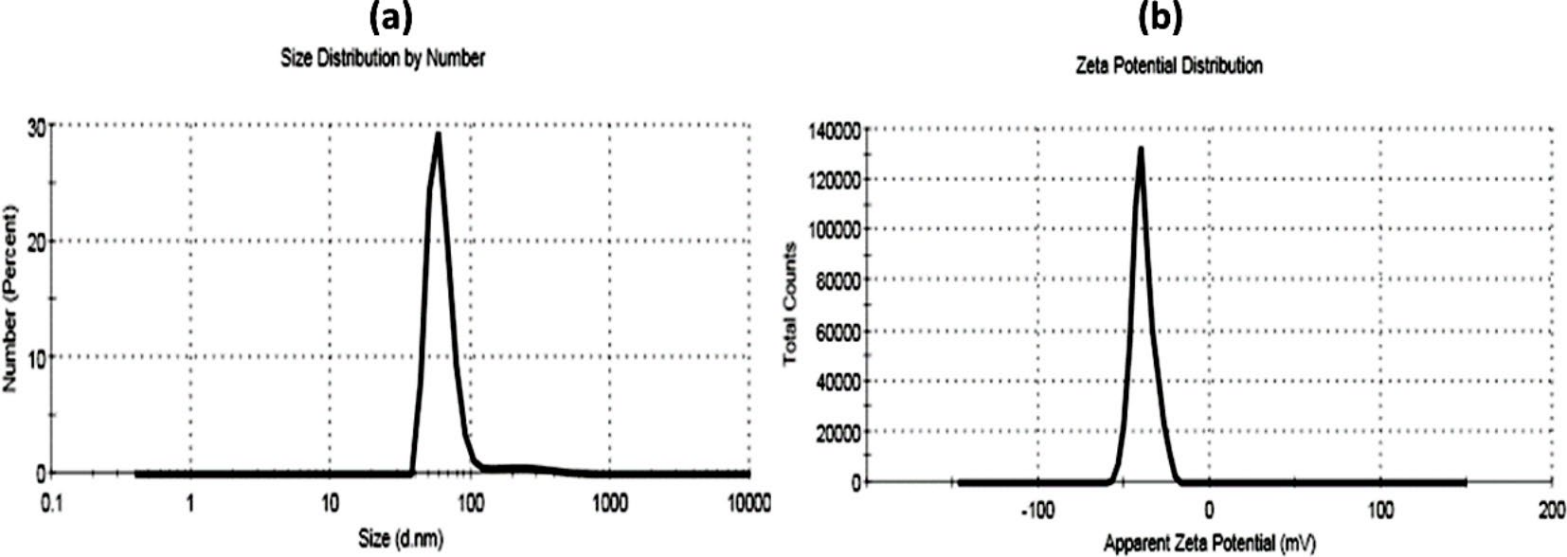
The particle size distribution by number (**a**) and Zeta potential value (**b**) of sample A using DLS technique.

**Fig. 3:**
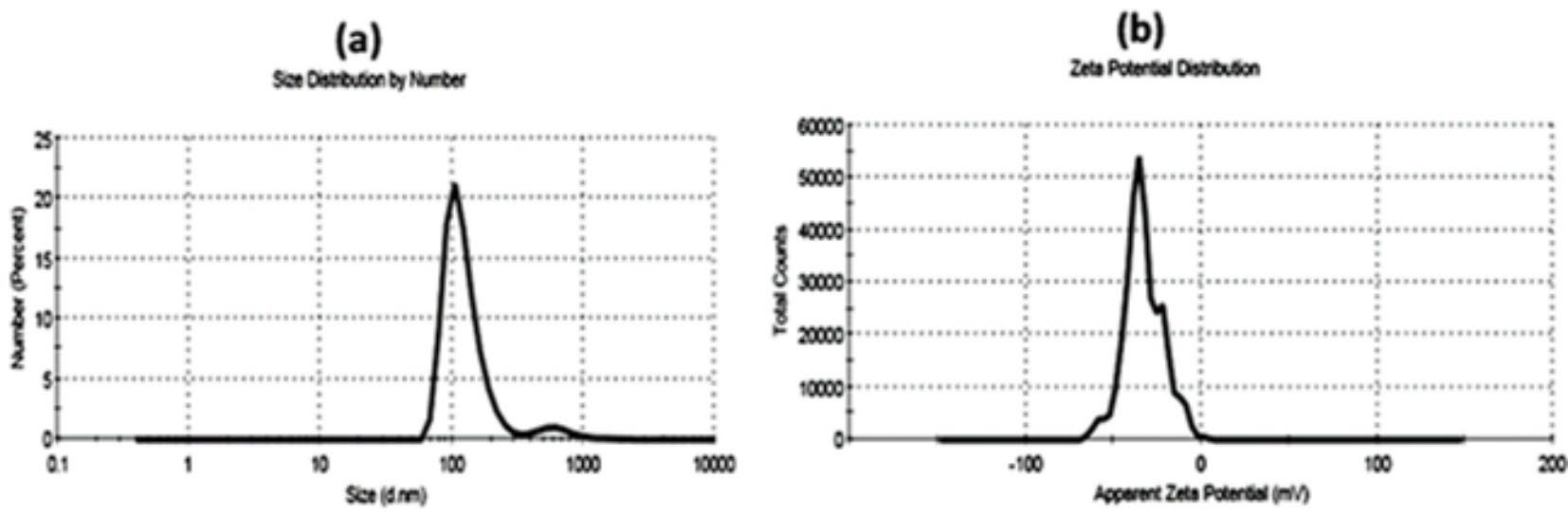
The particle size distribution by number (**a**) and Zeta potential value (**b**) of sample B using DLS technique.

The calculated values of the polydispersity index (PDI) for the prepared sample A was 0.57 and sample B was 0.6. The particle size distribution and surface charge of sample A and sample B measured by the dynamic light scattering (DLS) technique were represented in Figs. 2 and 3 respectively. As shown in the figures, both sample A (Fig. 2-a) and sample B (Fig. 3-a) exhibit size distribution by number of about 62 and 123 d.nm respectively. Zeta potential of sample A (Fig. 2-b) is -38.9 mV, while Zeta potential of sample B (Fig. 3-b) was − 32.8 mV.

#### Fourier transform infrared (FTIR)

**Fig. 4:**
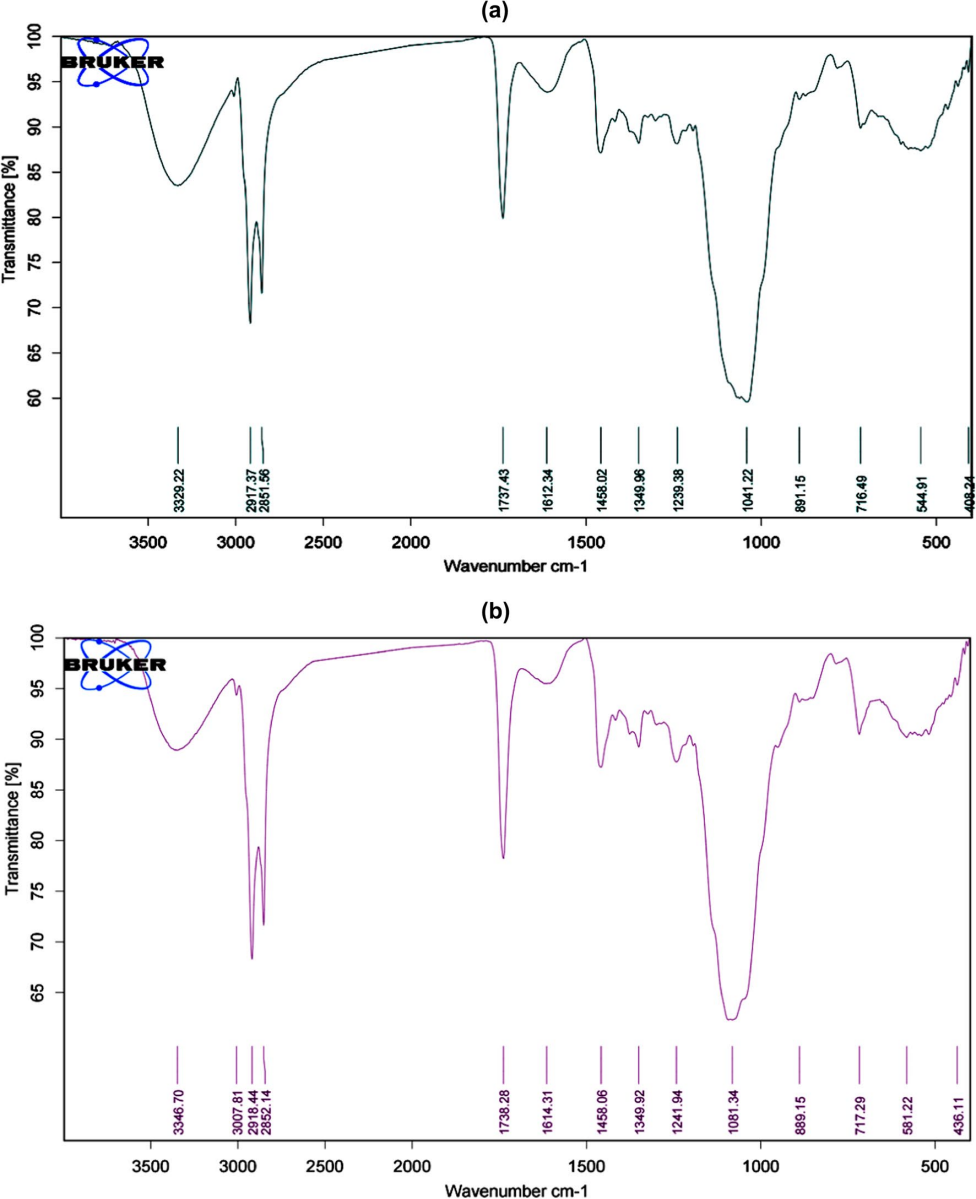
(**a**) FTIR spectra showing the active groups for sample A. (**b**) FTIR spectra showing the active groups for sample B.

The FTIR spectrum of sample A (Fig. 4a) and sample B (Fig. 4b) showed almost the same peaks; the absorption band at 3329 cm^− 1^ of sample A and the absorption band 3347 cm^− 1^ of sample B describ the stretching mode of hydroxyl (OH) group. The bands at 2917 cm^− 1^ and 2852 cm^− 1^ of sample A and the bands at 2918 cm^− 1^ and 2852 cm^− 1^ of sample B are characteristic for vibration modes of methylene (CH_2_) group. The band at 1737 cm^− 1^ of sample A and the band at 1738 cm^− 1^ of sample B is unique to the carbonyl (C = O) group. The band at 1612 cm^− 1^ of sample A and the band at 1614 cm^− 1^ of sample B corresponds to the alkene (C = C) group.Whereas, the two bands observed at 1458 cm^− 1^ and 1350 cm^− 1^ of both samples A and B point to methyl (CH_3_) and methylene (CH_2_) groups respectively. Finally, the stretching mode of ether (C-O-C) group is noted band at the absorbtion band 1041 cm^− 1^ of sample A and 1081 cm^− 1^ of sample B.

#### Thermogravimetric analysis (TGA)

**Fig. 5:**
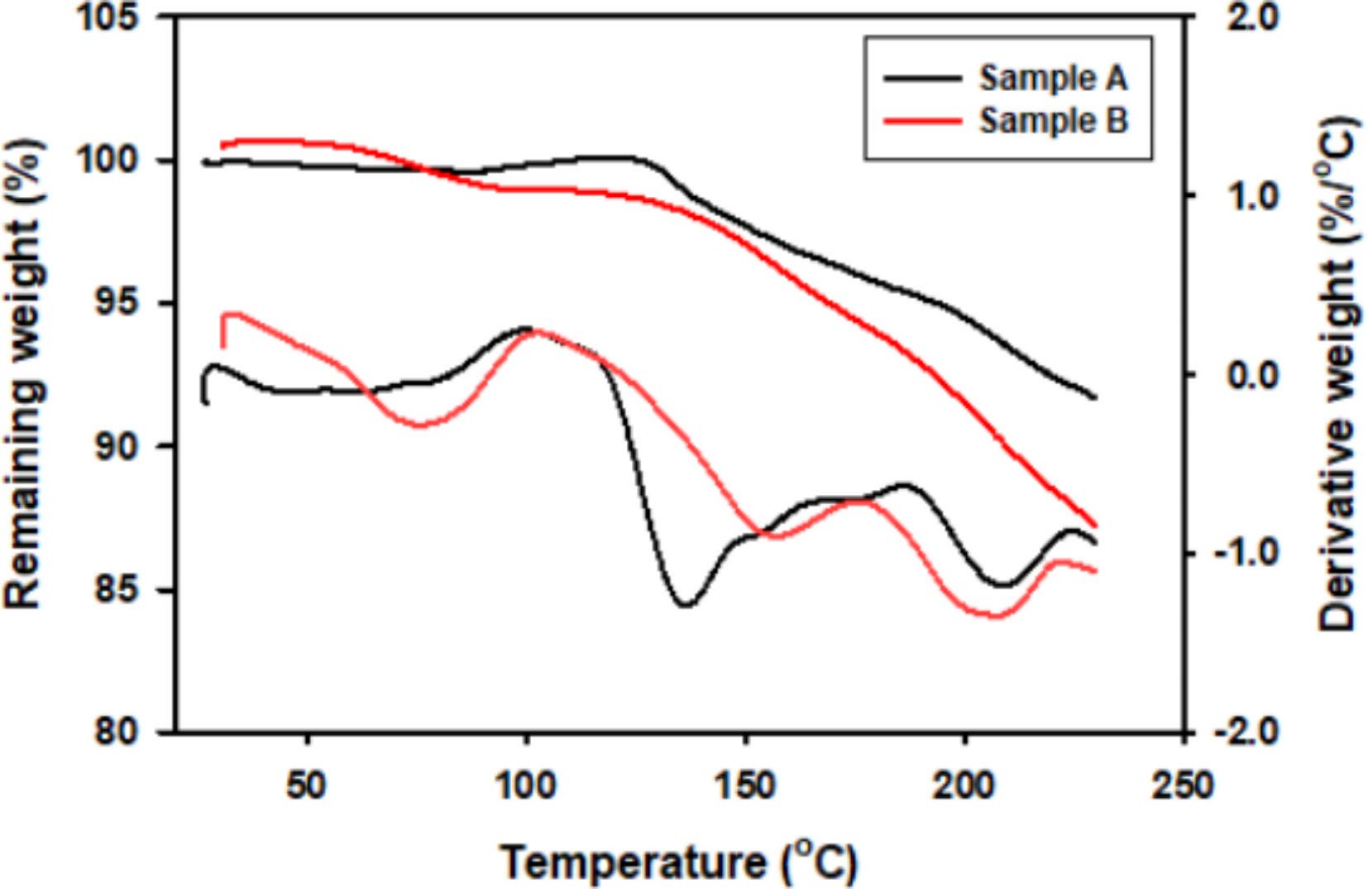
TGA curve for sample A and sample B showing a thermal stability until 207 °C and 202 °C, respectively.

The two samples A and B are tested until 225 °C to detect their thermal stability. Sample A shows a stable configuration until 207 °C because of the weight loss of the sample was approximately 8% (Fig. 5). While sample B shows stable configuration until 202 °C because of the weight loss of the sample was approximately 12% (Fig. 5).

### Free radical scavenging capacity of the treatment samples in vitro

**Fig. 6:**
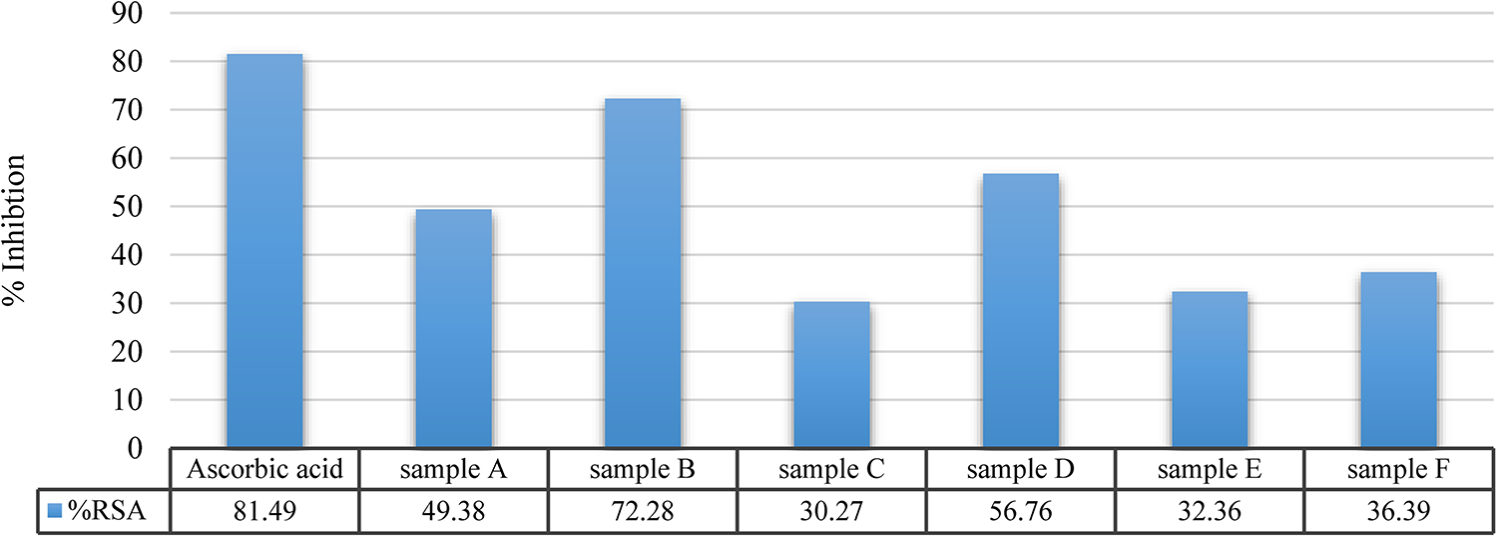
% inhibition of DPPH free radical of the treatment samples in comparison with ascorbic acid.

The free radical scavenging activity of the treatment samples A, B, C, D, E and F was measured by DPPH radical scavenging assay. In comparison with ascorbic acid, the % inhibition of DPPH free radical for ascorbic acid and samples A, B, C, D, E and F was 81.49%, 49.38%, 72.28%, 30.27%, 56.76%, 32.36% and 36.39% respectively (Fig. 6).

### Biochemical findings

#### Total testosterone

The present data revealed that there is a significant decrease (*p* < 0.05) in serum total testosterone level in BPA young-challenged group in comparison to young control group by 60.5%. On the other side, a significant increase (*p* < 0.05) in serum total testosterone level is recorded in nano young-treated group, free young-treated group and Vit.E young-treated group in comparison to BPA young challenged-group by 97.9%, 88.3% and 42.8% respectively. Additionally, there is a significant enhancement (*p* < 0.05) in serum total testosterone level in nano young-treated group and free young-treated group by contrast with Vit.E young-treated group by 38.6% and 31.9% respectively. Also, the present results indicated a significant reduction (*p* < 0.05) in serum total testosterone level in BPA aged-challenged group *versus* aged control group by 60.8%. Meanwhile, there is a significant increase (*p* < 0.05) in serum total testosterone level in nano aged-treated group, free aged-treated group and Vit.E aged-treated group contrary to BPA aged challenged-group by 109.6%, 92.6% and 39.3%, respectively. The present data also indicated that there is a significant enhancement (*p* < 0.05) in serum total testosterone level in nano aged-treated group and free aged-treated group in comparison to Vit.E aged-treated group by 50.5% and 38.3% respectively (Table 1).

**Table 1:** Impact of different treatments on serum total testosterone level in BPA-induced infertility in male rats.

Groups	Total testosterone (ng/ml)
Young control group	3.67 ± 0.023
Aged control group	3.44 ± 0.095
BPA young-challenged group	1.45 ± 0.197 ^a^ (-60.5%)
BPA aged-challenged group	1.35 ± 0.082 ^h^ (-60.8%)
Nano young-treated group	2.87 ± 0.318 ^bf^ (97.9%) (38.6%)
Nano aged-treated group	2.83 ± 0.058 ^im^ (109.6%) (50.5%)
Free young-treated group	2.73 ± 0.186 ^cg^ (88.3%) (31.9%)
Free aged-treated group	2.60 ± 0.081 ^jn^ (92.6%) (38.3%)
Vit.E young-treated group	2.07 ± 0.127 ^d^ (42.8%)
Vit.E aged-treated group	1.88 ± 0.015 ^k^ (39.3%)

^a^ Significant difference between young control group and BPA young-challenged group. ^b^ Significant difference between BPA young-challenged group and Nano young-treated group. ^c^ Significant difference between BPA young-challenged group and Free young-treated group. ^d^ Significant difference between BPA young-challenged group and Vit.E young-treated group. ^f^ Significant difference between Vit.E young-treated group and Nano young-treated group. ^g^ Significant difference between Vit.E young-treated group and Free young-treated group. ^h^ Significant difference between aged control group and BPA aged-challenged group. ^i^ Significant difference between BPA aged-challenged group and Nano aged-treated group. ^j^ Significant difference between BPA aged-challenged group and Free aged-treated group. ^k^ Significant difference between BPA aged-challenged group and Vit.E aged-treated group. ^m^ Significant difference between Vit.E aged-treated group and Nano aged-treated group. ^n^ Significant difference between Vit.E aged-treated group and Free aged-treated group.

#### Free testosterone

The current findings revealed that there is a significant drop (*p* < 0.05) in serum free testosterone level in BPA young-challenged group in comparison to young control group by 38.9%. On the other side, there is a significant enhancement (*p* < 0.05) in serum free testosterone level in nano young-treated group and free young-treated group in comparison to BPA young challenged-group by 40.5% and 28.4% respectively. Additionally, there is a significant elevation has been recorded (*p* < 0.05) in serum free testosterone level in nano young-treated group and free young-treated group *versus* Vit.E young-treated group by 37% and 25.2% respectively. The present results also indicated a significant reduction (*p* < 0.05) in serum free testosterone level in BPA aged-challenged group contrary to aged control group by 42.4%. Meanwhile, a significant augmentation (*p* < 0.05) in serum free testosterone level in nano aged-treated group, free aged-treated group and Vit.E aged-treated group by contrast with BPA aged challenged-group by 38.9%, 25.3% and 21.1% respectively has been observed (Table 2).

**Table 2:** Impact of different treatments on serum free testosterone level in BPA-induced infertility in male rats.

Groups	Free testosterone (pg/ml)
Young control group	1.90 ± 0.035 (15.15%)
Aged control group	1.65 ± 0.064
BPA young-challenged group	1.16 ± 0.032 ^a^ (-38.9%)
BPA aged-challenged group	0.95 ± 0.086 ^h^ (-42.4%)
Nano young-treated group	1.63 ± 0.033 ^bf^ (40.5%) (37%)
Nano aged-treated group	1.32 ± 0.017 ^i^ (38.9%)
Free young-treated group	1.49 ± 0.038 ^cg^ (28.4%) (25.2%)
Free aged-treated group	1.19 ± 0.072 ^j^ (25.3%)
Vit.E young-treated group	1.19 ± 0.134 (-37.3%)
Vit.E aged-treated group	1.15 ± 0.064 ^k^ (21.1%)

^a^ Significant difference between young control group and BPA young-challenged group. ^b^ Significant difference between BPA young-challenged group and Nano young-treated group. ^c^ Significant difference between BPA young-challenged group and Free young-treated group. ^f^ Significant difference between Vit.E young-treated group and Nano young-treated group. ^g^ Significant difference between Vit.E young-treated group and Free young-treated group. ^h^ Significant difference between aged control group and BPA aged-challenged group. ^i^ Significant difference between BPA aged-challenged group and Nano aged-treated group. ^j^ Significant difference between BPA aged-challenged group and Free aged-treated group. ^k^ Significant difference between BPA aged-challenged group and Vit.E aged-treated group.

#### Malondialdehyde (MDA)

The present data revealed that there is a significant rise (*p* < 0.05) in serum MDA level in BPA young-challenged group *versus* young control group by 78.8%. On the opposite side, a significant depletion (*p* < 0.05) in serum MDA level in nano young-treated group, free young-treated group and Vit.E young-treated group, as compared to BPA young challenged-group by 36.8%, 21.5% and 15.3% respectively, has been detected. Additionally, the data in present work denoted a significant decrement (*p* < 0.05) in serum MDA level in nano young-treated group contrary to free young-treated group by 19.5%. Also, the current study found that there is a significant decline (*p* < 0.05) in serum MDA level in nano young-treated group and free young-treated group in comparison to Vit.E young-treated group by 25.3% and 7.3% respectively. Furthermore, a significant elevation (*p* < 0.05) in serum MDA level in BPA aged-challenged group in comparison to aged control group by 85.9% has been demonstrated. However, a significant decrease (*p* < 0.05) in serum MDA level in nano aged-treated group, free aged-treated group and Vit.E aged-treated group in comparison to BPA aged challenged-group by 38.2%, 24.4% and 16.8%, respectively has been determined. Likewise, a significant reduction (*p* < 0.05) in serum MDA level in nano aged-treated group in comparison to free aged-treated group by 18.3% has been observed. Finally, the findings in present work demonstrated a significant diminution (*p* < 0.05) in serum MDA level in nano aged-treated group and free aged-treated group by contrast with Vit.E aged-treated group by 25.7% and 9.1% respectively (Table 3).

**Table 3:** Impact of different treatments on serum MDA level in BPA-induced infertility in male rats.

Groups	MDA (nmol/ml)
Young control group	2.45 ± 0.062
Aged control group	2.49 ± 0.107
BPA young-challenged group	4.38 ± 0.033 ^a^ (78.8%)
BPA aged-challenged group	4.63 ± 0.122 ^h^ (85.9%)
Nano young-treated group	2.77 ± 0.018 ^bef^ (-36.8%) (-25.3%) (-19.5%)
Nano aged-treated group	2.86 ± 0.058 ^ilm^ (-38.2%) (-25.7%) (-18.3%)
Free young-treated group	3.44 ± 0.038 ^cg^ (-21.5%) (-7.3%)
Free aged-treated group	3.50 ± 0.035 ^jn^ (-24.4%) (-9.1%)
Vit.E young-treated group	3.71 ± 0.084 ^d^ (-15.3%)
Vit.E aged-treated group	3.85 ± 0.031 ^k^ (-16.8%)

^a^ Significant difference between young control group and BPA young-challenged group. ^b^ Significant difference between BPA young-challenged group and Nano young-treated group. ^c^ Significant difference between BPA young-challenged group and Free young-treated group. ^d^ Significant difference between BPA young-challenged group and Vit.E young-treated group. ^e^ Significant difference between Nano young-treated group and Free young-treated group. ^f^ Significant difference between Vit.E young-treated group and Nano young-treated group. ^g^ Significant difference between Vit.E young-treated group and Free young-treated group. ^h^ Significant difference between aged control group and BPA aged-challenged group. ^i^ Significant difference between BPA aged-challenged group and Nano aged-treated group. ^**j**^ Significant difference between BPA aged-challenged group and Free aged-treated group. ^k^ Significant difference between BPA aged-challenged group and Vit.E aged-treated group. ^l^ Significant difference between Nano aged-treated group and Free aged-treated group. ^m^ Significant difference between Vit.E aged-treated group and Nano aged-treated group. ^n^ Significant difference between Vit.E aged-treated group and Free aged-treated group.

#### 8-hydroxy-2′-deoxyguanosine (8-OHdG)

The data represented in the present approach revealed that there is a significant elevation (*p* < 0.05) in serum 8-OHdG level in BPA young-challenged group in comparison to young control group by 84.4%. On the opposite hand, a significant decline (p < 0.05) in serum 8-OHdG level in nano young-treated group, free young-treated group and Vit.E young-treated group *versus* BPA young challenged-group by 42.8%, 34.5% and 24.3% respectively, has been found. Similarly, a significant decrement (p < 0.05) in serum 8-OHdG level in nano young-treated group in comparison to free young-treated group by 12.7% has been detected. Also, a significant drop (p < 0.05) in serum 8-OHdG level in nano young-treated group and free young-treated group as compared to Vit.E young-treated group by 24.5% and 13.5% respectively has been observed. Moreover, the present results noted a significant enhancement (p < 0.05) in serum 8-OHdG level in BPA aged-challenged group *versus* aged control group by 89%. Meanwhile, a significant suppression (p < 0.05) in serum 8-OHdG level in nano aged-treated group, free aged-treated group and Vit.E aged-treated group by contrast with BPA aged challenged-group by 42.1%, 36.9% and 22.7% respectively has been recorded. Likewise, the present findings indicated a significant inhibition (p < 0.05) in serum 8-OHdG level in nano aged-treated group and free aged-treated group when compared to Vit.E aged-treated group by 25.2% and 18.3% respectively (Table 4).

**Table 4:** Impact of different treatments on serum 8-OHdG level in BPA-induced infertility in male rats.

Groups	8-OHdG (ng/ml)
Young control group	6.41 ± 0.277
Aged control group	6.53 ± 0.509
BPA young-challenged group	11.82 ± 0.393 ^a^ (84.4%)
BPA aged-challenged group	12.34 ± 0.199 ^h^ (89%)
Nano young-treated group	6.76 ± 0.174 ^bef^ (-42.8%) (-24.5%) (-12.7%)
Nano aged-treated group	7.14 ± 0.182 ^im^ (-42.1%) (-25.2%)
Free young-treated group	7.74 ± 0.085 ^cg^ (-34.5%) (-13.5%)
Free aged-treated group	7.79 ± 0.093 ^jn^ (-36.9%) (-18.3%)
Vit.E young-treated group	8.95 ± 0.110 ^d^ (-24.3%)
Vit.E aged-treated group	9.54 ± 0.194 ^k^ (-22.7%)

^a^ Significant difference between young control group and BPA young-challenged group. ^b^ Significant difference between BPA young-challenged group and Nano young-treated group. ^c^ Significant difference between BPA young-challenged group and Free young-treated group. ^d^ Significant difference between BPA young-challenged group and Vit.E young-treated group. ^e^ Significant difference between Nano young-treated group and Free young-treated group. ^f^ Significant difference between Vit.E young-treated group and Nano young-treated group. ^g^ Significant difference between Vit.E young-treated group and Free young-treated group. ^h^ Significant difference between aged control group and BPA aged-challenged group. ^i^ Significant difference between BPA aged-challenged group and Nano aged-treated group. ^j^ Significant difference between BPA aged-challenged group and Free aged-treated group. ^k^ Significant difference between BPA aged-challenged group and Vit.E aged-treated group. ^m^ Significant difference between Vit.E aged-treated group and Nano aged-treated group. ^n^ Significant difference between Vit.E aged-treated group and Free aged-treated group.

#### Advanced glycation end products (AGEs)

Results of the current investigation revealed that there is a significant increase (*p* < 0.05) in serum AGEs level in BPA young-challenged group in comparison to young control group by 71.4%. On the other side, the current data showed that significant decrease (*p* < 0.05) in serum AGEs level in nano young-treated group, free young-treated group and Vit.E young-treated group *versus* BPA young challenged-group by 39.1%, 36.3% and 19.9% respectively has been noted. Additionally, a significant reduction (*p* < 0.05) in serum AGEs level in nano young-treated group and free young-treated group by contrast with Vit.E young-treated group by 23.9% and 20.5% respectively has been demonstrated. Furthermore, present results indicated a significant elevation (*p* < 0.05) in serum AGEs level in BPA aged-challenged group in comparison to aged control group by 74.4%. Meanwhile, a significant decrease (*p* < 0.05) in serum AGEs level in nano aged-treated group, free aged-treated group and Vit.E aged-treated group in comparison to BPA aged challenged-group by 38.9%, 34.9% and 17.6%, respectively has been recorded. Likewise, a significant drop (*p* < 0.05) in serum AGEs level in nano aged-treated group and free aged-treated group contrary to Vit.E aged-treated group by 25.8% and 20.9% respectively has been observed (Table 5).

**Table 5:** Impact of different treatments on serum AGEs level in BPA-induced infertility y in male rats

Groups	AGE (ng/ml)
Young control group	88.70 ± 0.404
Aged control group	89.83 ± 0.441
BPA young-challenged group	152.04 ± 2.125 ^a^ (71.4%)
BPA aged-challenged group	156.63 ± 3.317 ^h^ (74.4%)
Nano young-treated group	92.60 ± 1.701 ^bf^ (-39.1%) (-23.9%)
Nano aged-treated group	95.77 ± 0.433^im^ (-38.9%) (-25.8%)
Free young-treated group	96.83 ± 6.585 ^cg^ (-36.3%) (-20.5%)
Free aged-treated group	102.03 ± 6.718 ^jn^ (-34.9%) (-20.9%)
Vit.E young-treated group	121.75 ± 5.924 ^d^ (-19.9%)
Vit.E aged-treated group	129.00 ± 3.786 ^k^ (-17.6%)

^a^ Significant difference between young control group and BPA young-challenged group. ^b^ Significant difference between BPA young-challenged group and Nano young-treated group. ^c^ Significant difference between BPA young-challenged group and Free young-treated group. ^d^ Significant difference between BPA young-challenged group and Vit.E young-treated group. ^f^ Significant difference between Vit.E young-treated group and Nano young-treated group. ^g^ Significant difference between Vit.E young-treated group and Free young-treated group. ^h^ Significant difference between aged control group and BPA aged-challenged group. ^i^ Significant difference between BPA aged-challenged group and Nano aged-treated group. ^j^ Significant difference between BPA aged-challenged group and Free aged-treated group. ^k^ Significant difference between BPA aged-challenged group and Vit.E aged-treated group. ^m^ Significant difference between Vit.E aged-treated group and Nano aged-treated group. ^n^ Significant difference between Vit.E aged-treated group and Free aged-treated group.

### Histological observations

**Fig. 7:**
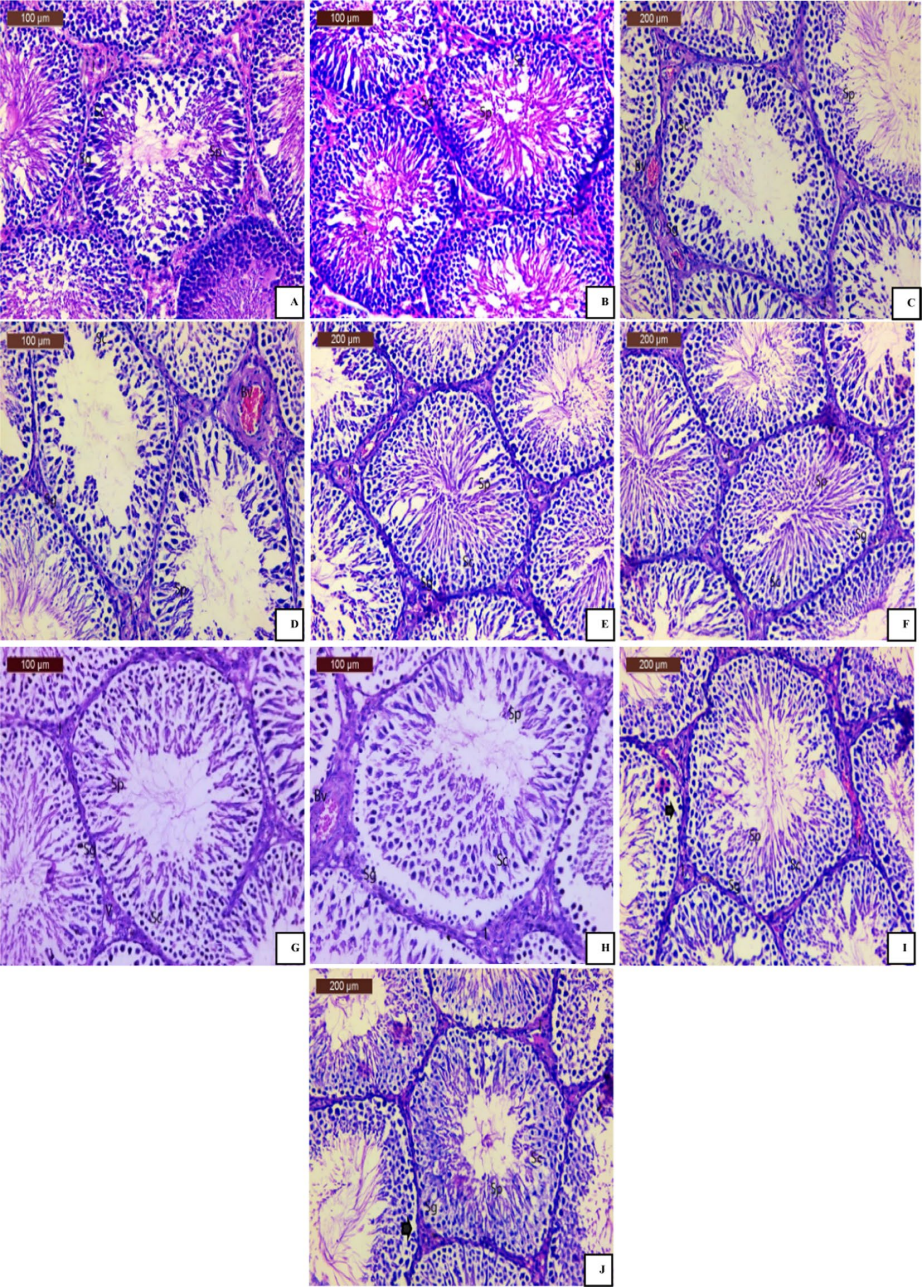
(**A**): Photomicrograph of the testicular section of young control group; (**B**): Photomicrograph of the testicular section of aged control group; (**C**): Photomicrograph of the testicular section of BPA young-challenged group; (**D**): Photomicrograph of the testicular section of BPA aged-challenged group; (**E**): Photomicrograph of the testicular section of Nano young-treated group; (**F**): Photomicrograph of the testicular section of Nano aged-treated group; (**G**): Photomicrograph of the testicular section of Free young-treated group; (**H**): Photomicrograph of the testicular section of Free aged-treated group; (**I**): Photomicrograph of the testicular section of Vit.E young-treated group; (**J**): Photomicrograph of the testicular section of Vit.E aged-treated group.

Histological examination of the testicular tissue section of rat in young control group (Fig. 7A) and that in aged control group (Fig. 7B) showed normal testicular architecture of seminiferous tubules that are lined with germinal epithelium which consists of cells that include different developmental stages of germ cells, namely (spermatogonia (Sg), spermatocytes (Sc) and spermatids (Sp)), spermatogonia (Sg) resting on thin basement membrane. The germinal epithelium (spermatogonia, spermatocytes and spermatids) separated by narrow interstitial space with Leydig cells (I).

The testicular tissue section of rat in BPA young-challenged group (Fig. 7) showed disorganized architecture of seminiferous tubules, degenerated germinal epithelium (deteriorated spermatogonia (Sg), spermatocytes (Sc) and spermatids (Sp)), with small dark nucleus and eosinophilic cytoplasm. Other tubules revealed disconnection of spermatogonia (Sg) from the basement membrane. The testicular blood vessels were congested (Bv), and the interstitial tissue appeared degenerated with loss of a large number of Leydig cells (I). Likewise, the testicular tissue section of rat in BPA aged-challenged group (Fig. 7D) showed disturbed architecture of seminiferous tubules, separation of spermatogonia (Sg) from the basement membrane, degenerated, necrotic changes of all germinal epithelium (spermatogonia (SG), spermatocytes (SC) and spermatids (Sp)), with small dark nucleus and eosinophilic cytoplasm. Markedly thickened congested blood vessel and degeneration of interstitial tissue (I) with hemorrhage were also observed (Bv).

Interestingly, the examination of testicular tissue section of rat in nano young-treated group (Fig. 7E) showed nearly normal testicular architecture, the seminiferous tubules with normally germinal epithelium (regular arrangement of spermatogonia (Sg) spermatocytes (Sc) and spermatids (Sp)), the spermatogonia (Sg) normally resting on thin basement membrane. The interstitial tissue was slightly degenerated (I) with mild congested blood vessels (Bv). Likewise, the testicular tissue section of rat in nano aged-treated group (Fig. 7F) showed the seminiferous tubules with apparently nearly normal architecture with nearly normal regular arranged germinal epithelium cells (spermatogonia (Sg), spermatocytes (Sc) and spermatids (Sp)), and the interstitial tissue was slightly degenerated (I) with mild congested blood vessels (Bv).

The examination of the testicular tissue section of rat in free young-treated group (Fig. 7G) showed apparently normal seminiferous tubules lined by multiple layers of spermatogenic cells (spermatogonia (Sg), spermatocytes (Sc) and spermatids (Sp)). The interstitial spaces contain apparently normal Leydig cells (I) with slight vacuolation (V). While the examination of the testicular section of rat in free aged-treated group (Fig. 7H) showed degeneration of spermatogenic cells, and some seminiferous tubules appeared with a well-organized germinal epithelium (spermatogonia (Sg), spermatocytes (Sc) and spermatids (Sp)). The spermatogonia (Sg) resting on thin basement membrane. Also, congested blood vessel (Bv), slight degeneration of interstitial tissue, detached germ layers in some seminiferous tubules, thickness of interstitial tissue (I) and few pyknotic interstitial cells of Leydig have been noted.

Histological examination of the testicular tissue section of rat in Vit.E young-treated group (Fig. 7I) showed degeneration of spermatogenic cells, but the germinal epithelium cells (spermatogonia (Sg), spermatocytes (Sc) and spermatids (Sp)) were more or less organized. However, the interstitial congestion was still observed. Meanwhile, the testicular tissue section of rat in Vit.E aged-treated group (Fig. 7J) showed a degeneration of spermatogenic cells, some seminiferous tubules appeared well organized, and spermatogonia (Sg) of germinal epithelium resting on thin basement membrane. Little intercellular spaces are noticed. Also, the interstitial space still wide with some leydig cells and eosinophilic material.

## Discussion

The TEM micrograph of the prepared dose of nanoemulsion of *Panax ginseng* dry extract for young adult rats (sample A) showed efficient distribution and obvious homogeneity. While, TEM micrograph of the prepared dose of nanoemulsion of *Panax ginseng* dry extract for aged adult rats (sample B) showed agglutination and aggregation clusters among the particles. These findings could be explained by the difference in dilution between the two samples as Petrochenko et al.^48^ referred that the concentration, referring to the number of particles observed per image, was notably reduced in the 10x diluted nanoemulsion samples in comparison to the undiluted ones. This is in consistent with the results of TEM micrograph of sample A that showed efficient distribution. Also, Wangensteen et al.^49^ reported that the TEM images from films with lower concentrations distinctly indicate the formation of smaller nanocrystals, characterized by many more boundaries per unit volume. Conversely, in films with higher concentrations, larger particles are observed with fewer boundaries per unit volume. On the other hand, both samples A and B showed almost spherical shape and this is in harmony with Md et al.^50^ findings which indicated that TEM imaging demonstrates the presence of uniform, spherical-shaped particles in the ginseng-loaded nanohydrogel, with no observable signs of particle aggregation. In addition, Zhang et al.^51^ observed that the surface morphology of the prepared ginsenoside compound K O-carboxymethyl chitosan nanoparticles (GK–OCMC NPs), as visualized by TEM, exhibits a uniform spherical shape with regular grain diameters and no evidence of aggregation.

The polydispersity index (PDI) is utilized to quantify the extent of non-uniformity of a size distribution of sample particles^52^. Essentially, it serves as an indicator of the distribution of different size populations within a sample. The PDI value ranges from 0.0, indicating a perfectly uniform sample in terms of particle size, to 1.0, representing a highly polydisperse sample with multiple particle size populations^53^. Sample A in current investigation had PDI value equal to 0.57 and exhibited a size distribution by number of about 62 d.nm, which indicated that that sample A had a homogenous and wide-size distribution in the prepared nanoemulsion. These findings are also confirmed by TEM micrograph as shown in Fig. 1a). On the other side, sample B had PDI value equal to 0.6 and exhibited a size distribution by number of about 123 d.nm, which denoted that the sample B had agglomerates and a narrow-size distribution in the prepared nanoemulsion. These findings were confirmed by TEM micrograph as shown in Fig. 1b as well.

Zeta potential quantifies the effective electric charge present on the surface of nanoparticles. The magnitude of the zeta potential offers insights into particle stability. A higher zeta magnitude indicates an electrostatic repulsion between particles in given sample, thus indicating increased stability^54^. Kumar and Dixit^55^ stated that if the zeta potential of the sample falls within the range of 0 to ± 5 thus, it indicates flocculation or coagulation in sample, within the range of ± 10 to ± 30, the sample shows incipient instability. For values ranging from ± 30 to ± 40, the sample exhibits moderate stability. If zeta potential falling between ± 40 to ± 60, the sample demonstrates good stability. Lastly, if the zeta potential range for the surface exceeds ± 60, the sample is deemed to possess excellent stability. In the present study, zeta potential recorded − 38.9 mV for sample A and − 32.8 mV for sample B. This can be explained as sample A has a high stability as it has larger zeta potential so the particles have a tendency to repel from each other and don’t aggregate as shown in TEM micrograph Fig. 1a. Whereas, sample B has smaller zeta potential, thus particles have a tendency to attract to each other again and form agglomerate as shown in TEM micrograph Fig. 1b.

The characteristic bands appeared in the FTIR of samples A and B in current approach showed the presence of hydroxyl (OH) group at 3329 cm^− 1^ and 3347 cm^− 1^ respectively, and this is in consistent with Hou^56^ findings that the characteristic band of ginsenosides in IR appeared approximately at wavelength 3380 cm^− 1^ representing OH stretch. Moreover, sample A showed the presence of bands at wavelengths 2917 cm^− 1^ and 2852 cm^− 1^, sample B showed the presence of bands at wavelengths 2918 cm^− 1^ and 2852 cm^− 1^ denoting for vibration modes of methylene (-CH_2_) group. These findings were proved by Owaid et al.^57^ and Choi et al.^58^ who reported the presence of two bands in FTIR of *Panax ginseng* at approximately 2923 cm^− 1^ and 2889 cm^− 1^ due to the asymmetric and symmetric vibration of methylene group respectively. The presence of carbonyl (C = O) group at 1737 cm^− 1^ of sample A, and at 1738 cm^− 1^ of sample B. In addition to the existence of alkene (C = C) group at 1612 cm^− 1^ of sample A and 1614 cm^− 1^ of sample B in FTIR are in agreement with Hou^56^ findings that IR of ginseng indicated the presence of band at approximately 1740 cm^− 1^ assigns to the stretching vibration of COOR group and band at 1620 cm^− 1^ clarifies C = C group. Moreover, the existence of bands at 1458 cm^− 1^ and 1350 cm^− 1^ of both samples A and B assigned to methyl (CH_3_) and methylene (-CH_2_) groups, as Liu et al.^59^ demonstrated that FTIR of ginseng shows two band approximately at 1458 cm^− 1^ and 1370 cm^− 1^ specific for the bending vibration of methyl and methylene groups. Furthermore, the bands appeared at 1041 cm^− 1^ and 1081 cm^− 1^ in FTIR of sample A and sample B respectively, assigned to ether (C-O-C) group comes in line with Choi et al.^58^ results that recorded the FTIR analysis of ginseng samples reveals the presence of numerous C-O-C groups, which are identifiable by characteristic bands within the spectral range of 1150 to 911 cm^− 1^.

Thermogravimetric analysis (TGA) is an analytical method utilized to evaluate the thermal stability of the material and assess its proportion of volatile components. This is achieved by monitoring the changes in weight as the sample undergoes controlled heating at a constant rate ^60^. The results of the present study denoted that sample A showed thermal stability until 207 °C and sample B showed thermal stability until 202 °C. Both samples showed a TGA curve profile of two weight loss phases; the first phase may be due to evaporation. Jiang et al.^61^ demonstrated that, the ginseng insoluble dietary fiber experiences evaporation at around 120 °C, leading to a slight weight loss of less than 2%. This observation suggests that this weight loss is likely attributable to the evaporation of moisture absorbed on the outer surface of the sample. While the second phase may be due to thermal dehydration as Hwang et al.^62^ reported that, upon heating ginseng at 150 °C, a significant reduction in weight occurs rapidly. This phenomenon is attributed to the additional dehydration of the glycosyl moiety at the C-3 and C-20 positions.

The DPPH assay is widely utilized as in vitro method for evaluating the plant extracts free radical scavenging activity ^63^. The higher value of inhibition percent (% inhibition), the greater antioxidant activity exhibited by the extract^64^. In our results, nanoemulsion of *Panax ginseng* dry extract samples A and B showed higher inhibition percent of 49.38% and 72.27% respectively. However, *Panax ginseng* dry extract free samples C and D showed lower inhibition percent of 30.26% and 56.75% respectively. These data fit with those of Rice-Evans et al.^65^ and Chang et al.^66^ findings that nano-grinding substantially enhanced the radical scavenging activity of ginseng in the DPPH assay as the nano-powdered ginseng demonstrating a higher inhibitory percentage compared to conventionally powdered ginseng. This can be elucidated by alterations in the molecular structure and positioning of polyphenol hydroxyl groups, which significantly impact the antioxidant activity of ginseng powder. Ultimately, this leads to modifications in radical scavenging activity by facilitating the donation of phenolic hydrogen and stabilizing the phenolic radical. Moreover, the inhibition percent (% inhibition) of sample B was higher than inhibition percent of sample A and the % inhibition of sample D was higher than % inhibition of sample C this may be due to different of concentration as El-Atawy et al.^67^ demonstrated that for all compounds, the % inhibition of DPPH increasing by increasing the concentration.

In in vivo experiment, oral administration of BPA caused significant reduction in both serum total and free testosterone levels in BPA young-challenged group and BPA aged-challenged group. This finding is in agreement with Nakamura et al.^68^ who reported that the administration of BPA at doses exceeding 100 mg/kg results in a reduction of plasma testosterone levels to approximately one-third in comparison to rats in control group. Biosynthesis of testosterone commences with the entry of cholesterol into the mitochondria this step is facilitated by the help of steroidogenic acute regulatory protein (StAR). Then inside the mitochondria, cholesterol is enzymatically converted into pregnenolone by the cholesterol side chain cleavage enzyme (P450scc)^68^. García et al.^69^ reported that elevated levels of ROS diminish the activity of P450scc, consequently hindering testosterone production in vitro. Also, Tsai et al.^70^ stated that hydrogen peroxide (H_2_O_2_) inhibits both basal and human chorionic gonadotropin-induced testosterone production from primary Leydig cells. This inhibition occurs through two mechanisms: firstly, by suppressing the activity of P450scc, and secondly, by reducing the expression of StAR in vivo. Additionally, studies have indicated that exposure to BPA induces oxidative stress in the testes of rodents^71^. As Olukole et al.^72^ noted that in the testes of rats treated with BPA, superoxide dismutases (SOD) were involved in the conversion of superoxide anion radicals into H_2_O_2_. Consequently, H_2_O_2_ accumulated in the testes due to its diminished elimination. Moreover, testosterone is synthesized through the canonical androgen production pathway, and it is playing a vital role in normal masculinization and the proper functioning of the testes. Within this canonical pathway, 17β-hydroxysteroid dehydrogenase type 3 (HSD17B3) is recognized as a pivotal enzyme involved in the biosynthesis of testosterone. It expressed solely within the testes and triggers the conversion of dehydroepiandrosterone (DHEA) into either androstenediol or androstenedione. Subsequently, both androstenediol and androstenedione undergo conversion into the biologically active androgen (testosterone) ^73^. At a low dosage, BPA exhibited a direct inhibitory effect on the activity of human and rat HSD17B3 enzymes by approximately 50% and 30%, respectively, leading to a decrease in testosterone synthesis^74,75^.

Currently, the elevated levels of serum total and free testosterone in nano young-treated group as well as nano aged-treated group are higher than their levels in Free young-treated group and free aged-treated group. These findings are in agreement with Kamel et al.^76^ and Linjawi^77^ results where the nanoparticles of *Panax ginseng* (PGNPs) in the small dose was better than free *Panax ginseng* (PG), as PGNPs markedly elevate serum levels of both total and free testosterone compared to free PG. Researchers observed that PGNPs have greater efficacy than free PG in enhancing symptoms of fertility in male rats by augmenting levels of male sex hormones. Linjawi^77^ proposed that the *Panax ginseng* nanoparticles enhance the efficacy of this botanical extract in reaching the target cells of the hypothalamic-pituitary-testicular axis, thereby enhancing male fertility. Furthermore, experimental results proved that the augmentative effect of nano-ginseng on the secretion of testicular hormones and the enhancement of sperm production^78^. It is known that ginseng or its derivatives have antioxidative properties through scavenging of free radicals, thereby inhibiting LPO^79^ and reducing production of H_2_O_2_ in rat testes^80^.

In the current work, groups treated with Vit.E displayed significant elevation in both serum total and free testosterone levels. Vit.E is a potent lipid-soluble antioxidant naturally occurring within cells, tends to accumulate in the membranes of mitochondria and endoplasmic reticulum. Its role involves safeguarding testicular cells against oxidative damage and LPO Gavazza and Catalá^81^ and Aydilek et al.^82^. Rats administered with 25 mg/kg of Vit.E exhibited an elevation in activity of antioxidant enzymes and concentrations of glutathione (GSH), alongside a reduction in LPO. In this context, Vit.E scavenges free radicals to maintain crucial cell membrane functions such as ion transport and membrane fluidity ^83^. Moreover, it safeguards molecules of testosterone from damage by impeding the polyunsaturated fatty acids (PUFA) oxidation^84^.

In current study, administration of BPA caused significant increase in serum MDA levels, and this observation aligns with the findings of Zhang et al.^85^, wherein exposure to BPA led to a notable increase in serum levels of MDA compared to the control group in vivo. Lipid peroxidation (LPO) refers to the oxidation of unsaturated fatty acids, with MDA being the resultant end product. MDA serves as a well-known parameter to substantiate the occurrence of oxidative stress ^42^. El Henafy et al.^86^ observed that BPA decreases the activity of testicular antioxidant enzymes [including glutathione reductase, superoxide dismutase, glutathione peroxidase (GPx) and catalase (CAT)], hence it significantly elevates LPO as well as MDA levels. Moreover, investigators asserted that BPA stimulates myriad oxidative damage within the testes of rats, leading to markedly elevated concentrations of MDA in both serum and sperm of male rats^87^. Thus, the assessment of MDA concentration could be valuable for evaluating male infertility ^88^.

Our findings showed a significant decrease in serum MDA levels in nano young-treated group and Nano aged-treated group more than Free young-treated group and Free aged-treated group. Studies have substantiated that *Panax ginseng*, known for its abundance of antioxidants, plays a vital role in regulating numerous pathways related to oxidative stress^89^. Ginsenosides, the primary active components found in *Panax ginseng*, exhibit potent antioxidant properties and mitigate oxidative stress by effectively scavenging ROS. Several in vitro and in vivo studies have proved that both *Panax ginseng* and its constituent ginsenosides augment the activity of antioxidant enzymes, consequently resulting in decreased levels of MDA^90^. Interestingly, ginseng berry silver nanoparticles (AgNPs) exhibited superior free radical scavenging activity in comparison to free ginseng berry. The heightened antioxidant activity observed in ginseng berry AgNPs might be attributed to the adsorption of bioactive compounds from ginseng onto the great surface area of spherical nanoparticles^32^. So this may be the reason for the better effect of ginseng nanoformulation than free ginseng.

In the present work, treatment with Vit.E brought about significant reduction in serum MDA levels both young and aged groups. In the study conducted by Malmir et al.^42^, the level of MDA exhibited a significant reduction in the BPA + Vit.E group compared to the BPA group. Through its vital role in augmentation of antioxidant enzyme activity, Vit.E can mitigate LPO, thereby ameliorating the detrimental alterations induced by BPA on testosterone levels, MDA concentrations and the testicular tissue. As noted by Babaei et al.^37^, the primary roles of Vit.E include inhibiting phospholipid membrane peroxidation and safeguarding cell membranes *via* its antioxidative properties.

In the current research, BPA induced a significant increase in serum 8-OHdG levels in BPA young challenged-group and BPA aged-challenged group. These findings are in harmony with those of Tiwari et al.^91^ which demonstrated that exposure to BPA raises plasma concentrations of 8-OHdG, augments LPO and reduces GSH activity. Omari et al.^92^ noted that 8-OHdG is among the primary forms of ROS, thus it is frequently employed as a biomarker to assess DNA oxidative damage. It represents one of the adducts resulting from the hydroxyl radical (HO^•^) assault on the deoxyguanosine residue^93^. The outcomes of in vivo investigation suggested that BPA augments the generation of HO^•^, which subsequently bind to DNA, resulting in the formation of 8-OHdG^94^.

Currently, the reduction of serum 8-OHdG levels in nano young-treated group as well as nano aged-treated group is more than the reduction of 8-OHdG in free young-treated group and free aged-treated group. *Panax ginseng* demonstrates robust antioxidant properties in both in vitro and in vivo studies, this is primarily attributable to its ginsenosides content^95^. Moreover, El-Banna et al.^96^ noted that ginsenoside Rg3 nanoparticles (Rg3-NPs) proved to be more efficacious than free ginsenoside Rg3 in mitigating DNA damage and dramatically reducing SOD as well as glutathione peroxidase (GPx) levels in mice. Scientists observed that utilizing liposomal nanovesicles as a nanocarrier for ginseng extract resulted in heightened intracellular antioxidant activity compared to administering free ginseng extract ^97^. Furthermore, the findings from both in vitro and in vivo studies conducted by Ullah et al.^98^ revealed a significant reduction in the levels of 8-OHdG in groups treated with red ginseng extract (RGE) and red ginseng oil (RGO). This suggests that both RGE and RGO possess the capability to protect DNA against oxidation and facilitate the recovery from DNA damage.

In our results, groups of rats (young and aged) treated with Vit.E showed significant decrement in serum 8-OHdG levels. Vitamin E plays a crucial role in scavenging free oxygen radicals such as perhydroxyl radical (HO_2_^•^) and hydroxyl radical (HO^•^) within the testes of adult rats ^99^. In Hassanien et al.^100^ study, Vit.E led to a notable reduction in serum levels of 8-OHdG, attributed to its known antioxidant properties. Furthermore, Oblette et al.^101^ demonstrated that the addition of Vit.E to cultures of fresh testicular tissues did not lead to an increase in the production of spermatozoa containing 8-OHdG adducts.

In the present work, groups administered BPA showed significant increase in serum AGEs level. Advanced glycation end products (AGEs) are the outcomes of non-enzymatic glycosylation of either endogenous or exogenous proteins^102^. They represent a dietary risk factor contributing to impaired spermatogenesis and reduced sperm quality in rodents. This is attributed to their pro-inflammatory and pro-oxidant properties, which significantly affect the sperm function ^103^. Advanced glycation end products (AGEs) bind to the AGE receptor (RAGE), thereby trigger the production of ROS and proinflammatory cytokines such as NF-κB^104^. Activation of NF-κB leads to the transcription of various target genes, including additional cytokines and the RAGE itself. One of the distinctive characteristic of RAGE-induced NF-κB activation is the establishment of a positive feedback loop, whereby the RAGE signal is sustained and reinforced^105^. The activation of NF-κB leads to the upregulation of RAGE expression, resulting in sustained stimulation and the promotion of signaling mechanisms that contribute to cellular damage^106^. Recently, Tekin and Çelebi^107^ reported a significant rise in NF-κB levels in testicular tissue following the administration of BPA compared to control groups in male rats. Hence, BPA indirectly elevates AGEs levels through NF-κB activation, subsequently leading to the upregulation of RAGE expression.

In the current approach, groups of rats (young and aged) treated with *Panax ginseng* either in nano form or free form exhibited significant decrease in serum AGEs levels. Ali et al.^108^ showed that *Panax ginseng* extract, particularly ginsenoside Rh2, inhibits AGEs formation and decreases fructosamine level. Additionally, it prevents oxidation of protein by reducing formation of protein carbonyl and modification of protein thiol groups. Thus, ginsenosides exhibit an effective role as anti-glycation agent for mitigating diabetic complications by inhibiting the formation of AGEs and oxidation-induced protein damage. The advantageous influence of *Panax ginseng* on oxidative damage induced by AGEs suggests that ginseng downregulates RAGE expression, consequently inhibits NF-κB activation^109^. Additionally, Kim^110^ discovered that ginsenosides Re and Rp1 directly inhibit the NF-κB signaling pathway.

Our results reported that the groups of young and aged rats treated with Vit.E experienced significant reduction in serum AGEs levels. Reckelhoff et al.^111^ reported that rats administered a high-Vit.E diet showed a downregulation in the expression of RAGE. This observation could potentially be attributed to the influence of Vit.E on the activation of NF-κB which subsequently impacts the expression of RAGE. Glauert^112^ noted that Vit.E inhibits NF-κB activation both in vitro and in vivo. The mechanisms through which Vit.E suppresses NF-κB activation likely pertain to its antioxidative properties. If NF-κB activation is triggered by oxidative stress, the antioxidant function of Vit.E is likely to be implicated. Additionally, Vit.E might impede the formation of lipoxygenase metabolites generated during LPO, which could otherwise activate NF-κB. Moreover, Vit.E blocks low-density lipoprotein (LDL) oxidation as LDL in their oxidized form has been observed to be stimulators of NF‐κB.

In the present investigation, photomicrograph of testicular tissue section of rat in BPA young-challenged group showed disorganized architecture of seminiferous tubules, degenerated germinal epithelium (spermatogonia, spermatocytes and spermatids), with small dark nucleus and eosinophilic cytoplasm, others tubules revealed disconnection of spermatogonia from the basement membrane. The testicular blood vessels were congested, and the interstitial tissue appeared degenerated with loss of a large number of Leydig cells. In addition, photomicrograph of testicular tissue section of rat in BPA aged-challenged group showed disturbed architecture of seminiferous tubules, present separation of spermatogonia from the basement membrane and degenerated germinal epithelium (spermatogonia, spermatocytes and spermatids), with small dark nucleus and eosinophilic cytoplasm, others tubules reveal reduction in the number of germinal epithelium cells, degeneration of interstitial tissue and thickened congested blood vessel. These data constitute Eid and Ahmed^113^ results which observed through histological examination of testicular tissue from the BPA-challenged group that there was a decrease in the typical organization of the germinal epithelium, characterized by mal-arrangement and abnormal cellular attachment. Furthermore, there was degeneration and necrosis in the spermatogenic cells. Also, Malmir et al.^42^ reported that the group exposed to a daily dose of 250 mg/kg of BPA showed a deterioration of the seminiferous tubule, disruption of spermatogenesis, and shedding of immature cells into the lumen. According to Nakamura et al.^68^ results, the examination of testicular tissue section from rat exposed to a 200 mg/kg dose of BPA revealed a decrease in the number of Leydig cells.

Currently, photomicrograph of the testicular tissue section of rat in Nano young-treated group showed apparently nearly normal testicular architecture, the seminiferous tubules with normally germinal epithelium (regular arrangement of spermatogonia, spermatocytes and spermatids), Interstitial tissue was slightly degenerated with mild congested blood vessels. Additionally, photomicrograph of the testicular tissue section of rat in Nano aged-treated group showed seminiferous tubules apparently nearly normal architecture with nearly normal regular arranged germinal epithelium (spermatogonia, spermatocytes and spermatids), the interstitial tissue was slightly degenerated with mild congested blood vessels. These results are in line with those of Kamel et al.^76^, where the administration of ginseng nanoparticles to rats notably mitigated the harmful effects of methotrexate (MTX) on testicular tissue and restored its normal histological structure. The ginseng nanoparticles effectively reversed impaired spermatogenesis, decreased disruption of seminiferous tubules, reduced detachment of germ cells, alleviated congestion, mitigated damage to Sertoli cells, and counteracted the effects of MTX on Leydig cells.

In present work, photomicrograph of the testicular tissue section of rat in Free young-treated group showed apparently normal seminiferous tubules lined by multiple layers of spermatogenic cells; spermatogonia, spermatocytes and spermatids. The interstitial spaces contain apparently normal Leydig cells with slight vacuolation. While, photomicrograph of the testicular tissue section of rat in Free aged-treated group showed degeneration of spermatogenic cells, some seminiferous tubules appeared with a well-organized germinal epithelium (spermatogonia, spermatocytes and spermatids). The spermatogonia resting on thin basement membrane. Also, congested blood vessel, slight degeneration of interstitial tissue, vacuolation, detached germ layers in some seminiferous tubules, thickness of interstitial tissue and few pyknotic interstitial cells of Leydig. These data come in line with those of with Kim et al.^114^ and Ali et al.^115^ that histological examination of the testicular tissue from rats treated with ginseng revealed the restoration of Leydig cells and spermatogenic layers. Additionally, there was a decrease in interstitial space, less basal membrane thickness, and a return to a typical arrangement of germinative cells within the seminiferous tubules.

Finally, photomicrograph of the testicular tissue section of rat in Vit.E young-treated group showed degeneration of spermatogenic cells. The germinal epithelium cells (spermatogonia, spermatocytes and spermatids) were more or less organized. However, interstitial congestion was still observed. Photomicrograph of the testicular tissue section of rat in Vit.E aged-treated group showed degeneration of spermatogenic cells, some seminiferous tubules appeared well organized and spermatogonia of germinal epithelium resting on thin basement membrane. Little intercellular spaces are noticed. Also, interstitial space still wide with some Leydig cells and eosinophilic material. These histological changes are in agreement with those of Malmir et al.^42^ which denoted that the group treated with BPA + Vit.E showed almost normal arrangement of germinal epithelium cells and regular spermatogenesis. In El Gharabawy et al.^116^ study, the examination of testicular tissue sections from rats treated with Vitamin E revealed the presence of typical seminiferous tubules, interspersed with a thin interstitial tissue containing Leydig cells and blood vessels. These tubules were encased by a consistent, delicate basement membrane and were lined with layers of Sertoli cells and spermatogenic cells (including spermatogonia, primary spermatocytes, spermatids, and spermatozoa). Therefore, the nanoemulsion of *Panax ginseng* dry extract proved to be the superior treatment. In particular, young rats treated with *Panax ginseng* dry extract nanoemulsion had the most promising results.

## Conclusion

According to our knowledge this is the first study for development of *Panax ginseng* dry extract nanoemulsion by oil in water (O/W) method. The evolved nanoemulsion formulization of *Panax ginseng* dry extract in the present study exhibited potential effect as male anti-infertility agent *via* normalizing the male sex hormones and regulating various pathways associated with oxidative stress. Importantly, the beneficial effect of nanoemulsion of *Panax ginseng* dry extract surpasses that of free *Panax ginseng* dry extract, resulting in a superior mitigation of BPA-induced male infertility. This is could be attributed to the small particles size and large surface area of the nanoemulsion.

## Data Availability

the data supporting the findings of this study are available within the paper. Should any raw data files be needed in another format they are available from the corresponding author upon reasonable request.
