# Publisher Correction: *Panax ginseng* nanoemulsion for counteracting male infertility via modulating sex hormones and oxidative stress in a rat model

**DOI:** 10.1038/s41598-025-94138-3

**Published:** 2025-03-27

**Authors:** Basma I. El-Shimi, Rafat M. Mohareb, Hanaa H. Ahmed, Rehab S. Abohashem, Khaled F. Mahmoud, Demiana H. Hanna

**Affiliations:** 1 Chemistry Department, Faculty of Science, Cairo University, Giza, Egypt; 2 Hormones Department, Medical Research and Clinical Studies Institute, National Research Centre, Dokki, Giza, Egypt; 3 Stem Cell Lab, Centre of Excellence for Advanced Sciences, National Research Centre, Dokki, Giza, Egypt; 4 Food Technology Department, Food Industry and Nutrition Research Institute, National Research Centre, Dokki, Giza, Egypt

Correction to: *Scientific Reports* 10.1038/s41598-024-79388-x, published online 25 November 2024

The original version of this Article contained an error in the following equation in the Methods section, under the subheading ‘Determination of free radical scavenging capacity in vitro using DPPH assay’.

$${\text{I}}({\backslash}{\%})=\frac{(Abs.\,of\,control\,-Abs.\,of\,sample\,or\,standard)}{Abs.\,of\,control}\times 100$$

now reads:

$${\text{I}}(\text{\%})=\frac{(Abs.\,of\,control\,-Abs.\,of\,sample\,or\,standard)}{Abs.\,of\,control}\times 100$$

In addition, under the subheading ‘Statistical analyses’,

$${\backslash}{\%}\,of\,change=\left(\frac{mean\,of\,treated\,group\,-mean\,of\,control\,group}{mean\,of\,control\,group}\right)\times 100$$

now reads:

$$\text{\%}\,of\,change=\left(\frac{mean\,of\,treated\,group\,-mean\,of\,control\,group}{mean\,of\,control\,group}\right)\times 100$$

Additionally, the legend of Figure 7 was incorrect.

“% inhibition of DPPH free radical of the treatment samples in comparison with ascorbic acid.”

now reads:

 “**(A):** Photomicrograph of the testicular section of young control group; **(B):** Photomicrograph of the testicular section of aged control group; **(C):** Photomicrograph of the testicular section of BPA young-challenged group; **(D):** Photomicrograph of the testicular section of BPA aged-challenged group; **(E):** Photomicrograph of the testicular section of Nano young-treated group; **(F):** Photomicrograph of the testicular section of Nano aged-treated group; **(G):** Photomicrograph of the testicular section of Free young-treated group; **(H):** Photomicrograph of the testicular section of Free aged-treated group; **(I):** Photomicrograph of the testicular section of Vit.E young-treated group; **(J):** Photomicrograph of the testicular section of Vit.E aged-treated group.”

The original Article has been corrected.

